# Large-Scale Saliva-Based Clinical Surveillance Enables Real Time SARS-CoV-2 Outbreak Detection and Genomic Tracking (Arizona, 2020–2023)

**DOI:** 10.3390/diagnostics15202663

**Published:** 2025-10-21

**Authors:** Steven C. Holland, ABCTL Diagnostic Testing and Sequencing Teams, Ian Shoemaker, Theresa Rosov, Carolyn C. Compton, Joshua LaBaer, Efrem S. Lim, Vel Murugan

**Affiliations:** 1 Virginia G. Piper Center for Personalized Diagnostics, Biodesign Institute, Arizona State University, Tempe, AZ 85281, USA; schollan@asu.edu (S.C.H.); shoemaker.ian@gmail.com (I.S.); trosov@cndlifesciences.com (T.R.); ccompto3@asu.edu (C.C.); jlabaer@asu.edu (J.L.); 2 ASU Biodesign Clinical Testing Laboratory, Biodesign Institute, Arizona State University, Tempe, AZ 85281, USA; 3 Communicable Diseases Agency, Singapore 307684, Singapore; efrem_lim@cda.gov.sg; 4 College of Health Solutions, Arizona State University, Phoenix, AZ 85004, USA; 5 Health Observatory, Arizona State University, Phoenix, AZ 85004, USA

**Keywords:** SARS-CoV-2, public health surveillance, saliva, next generation sequencing, gene target failure

## Abstract

**Background/Objectives:** Monitoring community health and tracking SARS-CoV-2 evolution were critical priorities throughout the COVID-19 pandemic. However, widespread shortages of personal protective equipment, the necessity for social distancing, and the redeployment of healthcare personnel to clinical duties presented significant barriers to traditional sample collection. **Methods:** In this study, we evaluated the feasibility of using self-collected saliva specimens for the qualitative detection of SARS-CoV-2 infection. Following confirmation of reliable viral detection in saliva, we established a large-scale surveillance program in Arizona, USA, to enable clinical diagnosis and genomic sequencing from self-collected samples. Between April 2020 and December 2023, we tested approximately 1.4 million saliva samples using RT-PCR, identifying 94,330 SARS-CoV-2 infections. Whole genome sequencing was performed on 69,595 samples, yielding 54,040 high-quality consensus genomes. **Results:** This surveillance approach enabled real-time monitoring of general infection trends that matched regional case counts. We monitored multiple wave-like introductions of viral lineages over the course of the pandemic. We identified three periods of S gene target failure on a commercial assay and assessed its ability to make fast, genotyping assignment during the pandemic (PPV = 0.98, 95% CI = 0.97–0.99; NPV = 0.94, 95% CI = 0.94–0.96). The co-location of clinical testing and sequencing capabilities within the same facility resulted in low turnaround time from the sample collection to the generation of sequencing data (median = 12 days, IQR: 9.0–19.75). **Conclusions:** Our findings support the use of self-collected saliva as a scalable, cost-effective, and practical strategy for infectious disease surveillance in future pandemics.

## 1. Introduction

The novel coronavirus SARS-CoV-2, the causative agent of COVID-19, was first observed in Wuhan, China in November 2019 [1]. On 30 January 2020, the World Health Organization (WHO) declared the SARS-CoV-2 virus and COVID-19 a public health emergency of international concern (PHEIC) [2]. On 5 May 2023, the WHO ended the PHEIC designation of SARS-CoV-2 and COVID-19 [3]. Over the three-year public health emergency, over 670 million cases of COVID-19 and over 6.8 million deaths are estimated to have occurred due to SARS-CoV-2 [4].

The emergence of novel SARS-CoV-2 lineages has raised public health concerns worldwide [5,6,7,8]. Arizona had the third reported case of COVID-19 in the USA [9]. Since evolution of SARS-CoV-2 would vary in their transmissibility or response to therapeutics [10,11], timely knowledge of the variant landscape in a region has important uses in planning public health response measures. The high transmissibility of SARS-CoV-2 and inadequate testing infrastructure available at the early stages of the pandemic created uncertainty to the status of the viral landscape in the USA [12]. The increasing need for healthcare workers in hospital and clinical environments also necessitated exploring sampling options that patients could perform independently.

To diagnose many respiratory infections, the recommended patient sample type is nasopharyngeal swab (NPS) or nasopharyngeal aspirate (NPA). These sample collection methods are often uncomfortable for patients and usually require collection by a trained healthcare worker, increasing the risk of virus transmission [13]. Because of these considerations and lower cost, saliva has been increasingly studied for its value in diagnosing respiratory infection [14,15,16,17].

Evolution of SARS-CoV-2 drove changes to diagnostic tests throughout the pandemic. On March 13, 2020, the TaqPath COVID-19 Combo Kit received Emergency Use Authorization (EUA) for the detection of SARS-CoV-2 in nasopharyngeal swabs, bronchoalveolar lavage, mid-turbinate swabs, nasal swabs, nasopharyngeal aspirate, and oropharyngeal swabs [18]. This assay contained primers and probes targeting the SARS-CoV-2 ORF1ab, S, and N genes but the oligo sequences were not publicly available. Continued discovery of lineages with mutations affecting test sensitivity at one gene locus, termed gene target failure (GTF) [19,20], led to the creation of the TaqPath COVID-19 Fast PCR Combo Kit 2.0 for use on saliva samples. While exact assay designs are not publicly available, the manufacturer describes that each gene (ORF1a, ORF1b, and N) is interrogated with multiple probes sharing the same fluorescent moiety [21]. With this scheme, multiple mutations within a gene are required for target failure. New target loci were also chosen in the updated assay, and the highly mutating S gene was no longer a gene target.

To address gaps in testing availability and the lack of available knowledge of viral dynamics of SARS-CoV-2 in Arizona, USA, we established and analyzed a multi-yearlong study using self-collected saliva samples. Here we report on the feasibility of saliva as a surveillance matrix, the prevalence of SARS-CoV-2 in Arizona, the longitudinal changes in SARS-CoV-2 variant composition, and the prevalence of S gene target failure (SGTF) in introduced lineages from April 2020 to December 2023. This work expands upon, and provides further context to, previously published studies using a limited number of samples within a narrow timeframe, that detail the diagnostic assay sensitivity [19], disease epidemiology [22], and Omicron variant transmission dynamics [23] of SARS-CoV-2 in Arizona that used this surveillance program as a source of sample recruitment or data comparison.

## 2. Materials and Methods

### 2.1. Participant Selection, Saliva Sample Kit Creation, Distribution, Collection, and Testing

Participants were recruited from partnered private organization employees, governmental organization employees, university students, and eventually their dependents and families. Recruitment comprised self-initiated testing and institution-selected testing. For invited testing, invitation frequency and selection method were determined by each institution independently. Depending on availability, the participants were able to choose a supervised collection site or to drop off a sample at an unsupervised site.

From 4 April 2020 until December 2021, supervised self-collected saliva drop-off sites were available. At these locations, trained staff and nurses were available to aid in sample collection and registration. At these locations, participant samples were maintained at 4 °C and were collected every day for RT-PCR testing.

For testing after February 2021, participants could self-collect saliva samples using saliva collection kits that contained a set of sample collection and registration instructions, an absorbent towel, alcohol wipe, a sample return bag, and a 6 mL collection tube (Micronic, Lelystad, The Netherlands) labeled with a 1 mL minimum sample volume and unique tube identifier (ID). Instructions informed participants to collect their saliva sample in the supplied tube, seal it in the sample return bag, and return the sample in a sample deposit box. Collection kits were placed near sample return locations and distributed to partner institutions.

Unsupervised drop-off sites were created where participants deposited their self-collected samples in sample deposit boxes. Sample deposit boxes varied but generally were a lockable container having a lid with a hole for sample return. Over the course of the survey period, approximately 40 deposit locations were used. The local conditions of the deposit bins varied, and climate-controlled locations were preferred, but the temperatures of the deposited samples were not regulated. Participants could drop off samples 24 h a day (dependent on location accessibility), and samples were usually collected twice daily.

Sample registration occurred when a participant electronically registered their collection tube ID to a custom HIPAA-compliant database and sample management software. Sample registration required participants to enter a 10-digit numeric tube ID and 5-digit alphanumeric sequence. The alphanumeric sequence was derived from the 10-digit sequence, so successful registration required both sequences to be correctly entered, thus minimizing human input error. Saliva samples had nucleic acids extracted and assayed for SARS-CoV-2 infection by RT-PCR. From April 2020 to October 2021, all collected saliva sample extracts were assayed using the TaqPath COVID-19 Combo Kit (Applied Biosystems; Waltham, MA, USA). After October 2021, saliva samples extracts were first analyzed using the TaqPath COVID-19 Fast PCR Combo Kit 2.0 (Applied Biosystems; Waltham, MA, USA) assay for diagnosis, and positive samples had their nucleic acids extracted and assayed using the TaqPath COVID-19 Combo Kit assay. Sample results were electronically returned after appropriate approval by quality control and assurance officers under Clinical Laboratory Improvement Amendments (CLIA) compliant protocols.

### 2.2. Affiliate Organization-Invited Testing Programs

Partnered organizations with invited testing programs were made aware of their organization’s test positivity results, but it is unknown to what extent organization policies or behaviors were modified in response. Most organizations participated in surveillance with one-off, or infrequent, testing programs. However, from May 2020 through February 2022, one organization maintained a long-term invited testing program with participant demographics like the self-initiated testing population. Weekly emails were sent to a randomly selected subset of organization affiliates inviting them to test in the following week. During discovered or suspected outbreak periods, no modifications to randomization or selection method are known to have been made. Participants invited to test could still participate in the self-initiated surveillance program at a rate and cadence of their choosing.

### 2.3. Limit of Detection and Heat Stability Testing of SARS-CoV-2 in Saliva

Heat-inactivated SARS-CoV-2 virus particles were obtained from ATCC (Catalog #VR-1986HK; Manassas, VA, USA). Three aliquots of virus particles were diluted to 100,000 copies/µL in a pool of SARS-CoV-2 negative saliva from 10 individuals and diluted to 3.1 copies/µL using 2-fold serial dilutions. Sample nucleic acids were extracted using the MagMAX assay and assayed for detection of SARS-CoV-2 using the TaqPath COVID-19 Combo Kit in triplicate. For confirmation of limit of detection, 24 samples at 100,000,000 SARS-CoV-2 virus particles/µL were generated and serially diluted to 12,500 particles/mL. Sample nucleic acids were extracted using the MagMAX assay and tested for SARS-CoV-2 detection using the TaqPath COVID-19 Combo Kit at concentrations 1×, 2×, 4×, 8×, 16×, and 24× the limit of detection. Heat stability assays of SARS-CoV-2 in saliva were measured in three replicates.

### 2.4. Paired NPS and Saliva Sample Collection

Paired saliva and NPS were collected from 148 asymptomatic individuals. Samples were recruited from voluntary study participants that were asked to give an NPS sample in addition to their saliva sample. Paired sample collection started in April 2020 and proceeded through May of 2020 until 20 NPS samples tested positive for SARS-CoV-2. NPS were collected by trained nurses and stored in 500 µL viral transport medium (VTM). Saliva samples were self-collected by each participant. NPS and saliva samples were collected within 30 min of each other. NPS and saliva samples had their nucleic acids extracted and assayed for the presence of SARS-CoV-2 using the TaqPath COVID-19 Combo Kit as described above.

### 2.5. Nucleic Acid Extractions

Nucleic acid extractions were performed using the MagMAX Total RNA Isolation Kit (Invitrogen, Waltham, MA, USA) using a KingFisher Flex (Thermo Scientific, Waltham, MA, USA). Briefly, 200 µL of saliva sample was added to 10 µL of total nucleic acid magnetic beads and 265 µL Binding Solution. For NPS, 400 µL of NPS storage solution was added to 20 µL of total nucleic acid magnetic beads and 530 µL Binding Solution. Sample binding mixes, required buffers, and wash solutions were prepared in 96-well format using a Biomek i7 automated liquid handler (Beckman Coulter Life Sciences, Indianapolis, IN, USA). With the MagMAX instrument loaded with wash solutions, diluted TURBO DNase, and elution buffers as directed by the manufacturer’s protocol, the ‘MagMAX Total’ program was run on the KingFisher Flex. Saliva and NPS samples were eluted to a final volume of 100 µL. Aliquots of nucleic acid extracts were used for immediate testing and the remainder stored at −80 °C.

### 2.6. RT-PCR Assays

Nucleic acid extracts run on the TaqPath COVID-19 Combo Kit (Applied Biosystems; Waltham, MA, USA) were assayed following the manufacturer’s instructions for 384-well format. Assay plates were prepared using a custom automation method on a Biomek i7 automated liquid handler. For saliva samples, 11 µL of nucleic acid extract was used as sample input and combined with 11 µL reconstituted Reaction Mix. For NPS, 5 µL of nucleic acid extract was used as sample input and combined with 20 µL reconstituted Reaction Mix. The RT-PCR cycling reactions were performed and analyzed on a QuantStudio 7 Flex (Applied Biosystems; Waltham, MA, USA) instrument with the following cycle conditions: 1 cycle of 25 °C for 2 min, 53 °C for 10 min, 1 cycle of 95 °C for 2 min, and 40 cycles of 95 °C/60 °C for 3 s/30 s. Assay interpretations were performed as per manufacturer’s recommendations.

Saliva samples run on the TaqPath COVID-19 Fast PCR Combo Kit 2.0 (Applied Biosystems; Waltham, MA, USA) were assayed following the manufacturer’s instructions for 384-well format. Assay plates were prepared using a custom automation method on a Biomek i7 automated liquid handler. Briefly, 22 µL of saliva was added to 22 µL SalivaReady and incubated for 5 min at 62 °C and then 5 min at 92 °C. Then 14 µL of the SalivaReady reaction was combined with 6 µL of reconstituted Assay Reaction Mix. The RT-PCR cycling reactions were performed and analyzed on a QuantStudio 7 Flex (Applied Biosystems; Waltham, MA, USA) instrument with the following cycle conditions: 53 °C for 5 min, 1 cycle of 85 °C for 5 min, 1 cycle of 95 °C for 2 min, and 40 cycles of 95 °C/62 °C for 1 s/30 s. Assay interpretations were performed as per the manufacturer’s recommendations.

Samples were marked invalid if marked by analysis software (e.g., poor RNase P amplification) or if saliva volumes were insufficient for testing, broken, or empty. A sample was determined to have SGTF if difference between the S gene C_t_ value and the mean C_t_ values of ORF1ab and N loci was greater than 3 (i.e., [S − mean (ORF1ab, N)] > 3). For computation purposes, undetected targets were assigned a C_t_ value of 40.

### 2.7. Participant Demographics Curation

During online registration, participants were asked to self-identify their sex, race, and ethnicity. Participants were also asked to enter their birthday. For each participant’s sex, race, and ethnicity, categorization of a participant was based on concordance of all of a patient’s registered samples. To be included as part of a cohort, all of a participant’s samples must have been registered with the same categorization (i.e., Male, Female, Unknown) or provided no response on other sample registrations. If a conflicting answer was provided on at least one tube, then the participant was assigned ‘Invalid or no answer’ for that categorization. For patient age at first test, the birthdate entered on the first registered sample was used to calculate the participant’s age. Ages exceeding 113 years were assigned ‘Invalid or no answer.’

### 2.8. Next Generation Sequencing of SARS-CoV-2

Sequencing facilities were in the same building where the diagnostic testing was performed. Whole viral genome sequencing was attempted on nearly all saliva samples testing positive for SARS-CoV-2 via RT-PCR after January 2021. NGS libraries were built on nucleic acid extracts using the COVIDSeq Test (Illumina, San Diego, CA, USA) reagents and IDT (Coralville, IA, USA) ARTICv3, ARTICv4, or ARTICv4.1 [24] primer sets. Libraries were prepared for sequencing using a custom automation method on a Biomek i7 liquid handler. Libraries were sequenced on an Illumina NextSeq2000 using 2 × 150 paired end reads using standard Illumina sequencing reagents and flow cells in P2 or P3 run sizes (Illumina 20046813 and 2046810). Reads were quality trimmed, filtered, and mapped using a custom pipeline. Over the course of the surveillance period, software algorithms were kept up to date at the time of a sample’s analysis and default parameters were used unless noted. Reads were trimmed of adapter sequences using trim-galore [25] (a wrapper for Cutadapt [26] and FastQC [27]) and aligned to the Wuhan-Hu-1 reference genome (Genbank MN908947.3) using the Burrows-Wheeler aligner, BWA-MEM (version 0.7.18-r1243) [28]. Primer sequences were removed from reads using iVAR (version 1.4.2; arguments: -e, -q 30) [29] software. Consensus genomes were created at positions with a minimum depth of 10 reads and a base call consensus threshold of 60% using samtools mpileup (version 1.17, arguments: -d 0, -AB, -aa, -Q 0) and iVAR consensus (version 1.4.2; arguments: -t 0.60, -m 10, -n N) [30]. Calculated ambiguities are the percentage of called bases not receiving an A, G, T, or C nucleotide base call. Lineage assignments were made using pangolin (current through version 4.3.1) [31], and validated using VADR (current through version 1.41.; arguments: --glsearch, -s, -r, --nomisc, --lowsim5seq 2, --lowsim5ftr 2, --lowsim3seq 2, --lowsim3ftr 2, --alt_pass peptrans, --alt_fail lowscore,fstukcft,insertnn,deletinn) [32]. Samples passing Pangolin QC checks, receiving a PANGO lineage, and having a mean genome coverage >20 were deposited to GISAID.

### 2.9. Statistical Analysis

LOWESS/LOESS regression was calculated using GraphPad Prism software (Version 10.6; Insight Partners, New York City, NY, USA). Cross-correlation and autocorrelation calculations, and Wilson’s confidence interval calculations were performed using RStudio software (version 2025.05.0).

## 3. Results

### 3.1. Saliva Is a Stable and Reliable Sample Matrix for SARS-CoV-2 Testing

To test the feasibility of using saliva as a SARS-CoV-2 sample collection and extraction matrix, we first assayed the limit of detection on pooled, SARS-CoV-2 negative saliva samples with known quantities of spiked-in, heat inactivated SARS-CoV-2 virus particles (Table 1). Using a preliminary sample size of 3 samples per concentration, we obtained positive results for SARS-CoV-2 in all 3 samples down to 12,207 virus particles/mL. In order to confirm using 12,500 as our limit of detection, we expanded the sample size from 3 to 24 samples for a series of low virus concentrations. We successfully detected SARS-CoV-2 in all samples (24/24) at 1×, 2×, 4×, 8×, 16×, and 32× the lower limit of detection (Table 1). The ORF1ab and S gene loci appeared to be slightly more sensitive than the N gene locus, with consistently lower C_t_ values and detecting SARS-CoV-2 down to 6103 particles/µL in one sample. Limit of detection analysis of NPSs resulted in a lower limit of detection of 1000 virus particles per milliliter of saliva (Supplemental Table S1). Factoring in the higher limit of detection and increased nucleic acid extraction input volume required (11 µL in a 22 µL reaction for saliva and 5 µL in a 25 µL reaction for NPS), we found that detection of SARS-CoV-2 in NPS outperforms detection in saliva.

**Figure 1:**
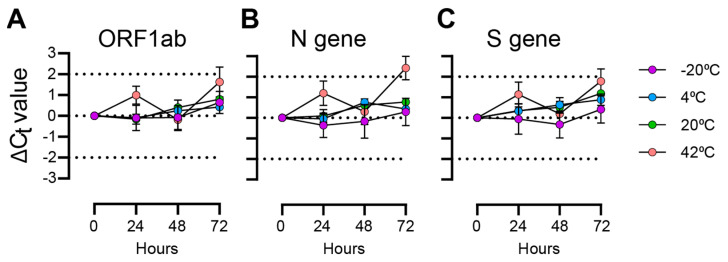
TaqPath C_t_ differences for saliva samples with spiked-in SARS-CoV-2 particles incubated over 72 h. Plotted values are C_t_ differences from 0 h measurement. Mean and standard deviations of 3 replicates are shown. (**A**) ORF1ab gene primer/probe target; (**B**) N gene primer/probe target; (**C**) S gene primer/probe target.

With self-collection, there are often delays between saliva collection and sample processing, so we tested SARS-CoV-2 virus particle detection at different times up to 72 h at four storage temperatures (Figure 1). At the ORF1ab locus, C_t_ values deviated by less than 1 C_t_ over 72 h for the −20 °C, 4 °C, and 20 °C samples. There were greater deviations observed in samples incubated at 42 °C, but differences to the initial C_t_ values stayed within 2 C_t_ values. Slightly greater C_t_ deviations were observed at the N gene and S gene loci. Overall, the differences we observed at the −20 °C and 4 °C temperatures deviated less than those incubated at 20 °C, and the samples incubated at 42 °C showed the greatest deviations.

Finally, we wanted to compare the concordance of saliva-based SARS-CoV-2 testing to ‘gold-standard’ NP swab SARS-CoV-2 testing (Table 2). We tested 148 paired NP and saliva samples for SARS-CoV-2 (prevalence = 20/148, 14%) using the TaqPath Combo Kit (Applied Biosystems; Waltham, MA, USA) assay. There was 95% positive concordance (19/20 samples; 95% Wilson’s CI: 76.4–99.1%) and 99.2% negative concordance (127/128 samples; 95% Wilson’s CI: 95.7–99.9%) between the two sampling methodologies. Sensitivity and specificity of saliva testing were 95.0% (95% CI 76.4–99.1%) and 99.2% (95% CI 95.7–99.9%), respectively. Positive and negative predictive values were 95.0% (95% CI 76.4–99.1%) and 99.2% (95% CI 95.7–99.9), respectively. The accuracy of saliva testing was 98.7% (95% CI 95.2–99.6%) and Cohen’s κ was 0.94, indicating almost perfect agreement between results.

With the finding that SARS-CoV-2 could be stably detected in saliva with good concordance to NPS testing, we continued with large-scale self-administered saliva collection and RT-PCR SARS-CoV-2 clinical surveillance.

### 3.2. Participant Demographics of Saliva-Based COVID-19 Surveillance

From 2 April 2020 to 31 December 2023, a total of 1,434,873 SARS-CoV-2 RT-PCR assays were performed on participant-collected saliva samples (Table 3). Over the surveillance period, 94,330 (6.6%) samples tested positive for SARS-CoV-2 and 10,922 (0.8%) samples were invalid. Most samples were collected from three Arizona counties: Maricopa County (74.5%), followed by Coconino County (15.8%), and Pima County (6.3%), with the remaining samples (3.2%) coming from other counties. There were 366,681 unique participants who provided saliva samples (Table 4). Slightly more participants identified as Male (50.7%) than Female (48.2%). Most participants identified as White (55.2%), but a large portion of participants provided an invalid answer or no answer (16.2%). A sizable portion of participants identified Hispanic or Latino ethnicity (21.9%). Most participants were aged 18–21 (21%) at the time of their first collection, indicating that college-aged students have a high representation in the participant set.

### 3.3. Self-Administered Saliva Collection Surveillance Reflects Community COVID-19 Epidemiology

**Figure 2:**
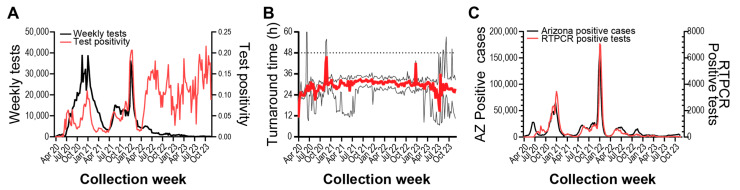
SARS-CoV-2 RT-PCR testing surveillance from April 2020 to November 2023. (**A**) Left axis: weekly RTPCR tests performed. Right axis: weekly test positivity, (Positive tests/Total tests). (**B**) Red line shows median weekly test result turnaround time. Lower and upper black lines show Q1 and Q3 values, respectively. Dashed line shows desired 48-h turnaround time. (**C**) Left axis: weekly Arizona SARS-CoV-2 case count. Right axis: surveillance program weekly RT-PCR positive samples.

Testing frequency was high during the initial surveillance period through 2022 but was greatly diminished throughout 2023 (Figure 2A). Generally, total testing was low in the summer months and higher during the academic school year. Test administration peaked in December 2020 and experienced another large rise in December 2021. Positivity rate fluctuated over the surveillance period, with peak positivity during January 2022. Interestingly, we observed that after July 2022, the positivity rate remained elevated compared to previous rates.

The median turnaround time from participant sample registration to electronic delivery of RT-PCR results was 29.2 h, and weekly median turnaround time stayed within 48 h (Figure 2B). We wanted to determine if our surveillance strategy and response time were indicative of SARS-CoV-2 infection in the broader community, so we compared our weekly positive test counts to Arizona COVID-19 case counts (Figure 2C). We found that trends between the two metrics were highly correlated, with our surveillance program lagging Arizona test positivity by approximately one week (Supplemental Figure S1A, r = 0.955, 95% CI: 0.940–0.966). Auto-correlation analyses also suggest a year-long periodicity to testing positivity and positive case counts in Arizona (Supplemental Figure S1B). Taken together, we observed that the diversity and responsiveness of our surveillance testing strategy sufficiently captured the local region’s epidemiological status.

### 3.4. Randomly Selected Test Participation Detected Infection Outbreaks

**Figure 3:**
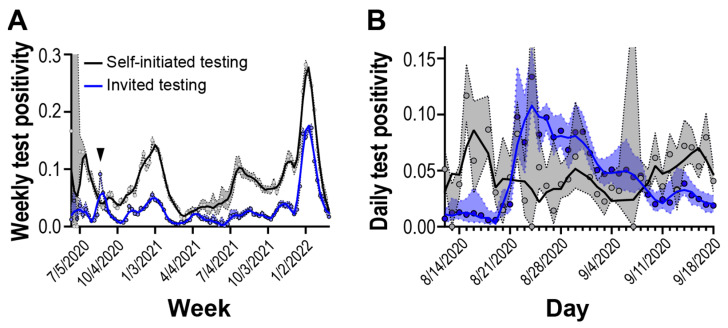
Test positivity rate of self-initiated and invited testing cohorts. Solid lines are 2nd order smoothing functions across 3 neighbor data points. Blue and gray shaded regions reflect 95% confidence intervals. (**A**) Weekly test positivity during the surveillance period. Arrow shows outbreak period. (**B**) Daily test positivity around outbreak period.

Our surveillance program comprised community participants self-initiating testing as well as participants invited to test by an affiliate organization (e.g., school, employer), so we wanted to compare how positivity between self-initiated testing compared to a population randomly selected and invited to test. Through May 2020 and February 2022, we compared test positivity for one organization-implementing randomized, invited testing to individuals self-initiating testing from the same geographic area. We observed that test positivity for self-initiated testing was generally higher than invited testing but followed similar upward and downward trends (Figure 3A, Supplemental Figure S2).

Starting on 22 August 2020, we observed a period of 17 days where the invited testing cohort displayed test positivity higher than the self-initiated testing cohort (Figure 3B). It took approximately 40 days for test positivity in the randomly selected invited cohort to return to pre-elevated levels. Overall, we observed that randomly selected invited testing can detect outbreaks not captured in self-initiated sampling strategies.

### 3.5. Saliva Sample Collection Enabled Whole Viral Genome Sequencing of Clinical Positives for Genomic Epidemiology Investigations

To monitor the introduction of VOCs and the emergence of novel mutations within circulating variants, whole viral genome sequencing of RT-PCR positive participant samples was performed. Over the surveillance period, sequencing of 69,595 saliva samples was attempted and 54,040 consensus genomes received sufficient coverage (median depth = 2474, IQR = 571–4790) for PANGO designation and were submitted to the GISAID database (Table 3).

**Figure 4:**
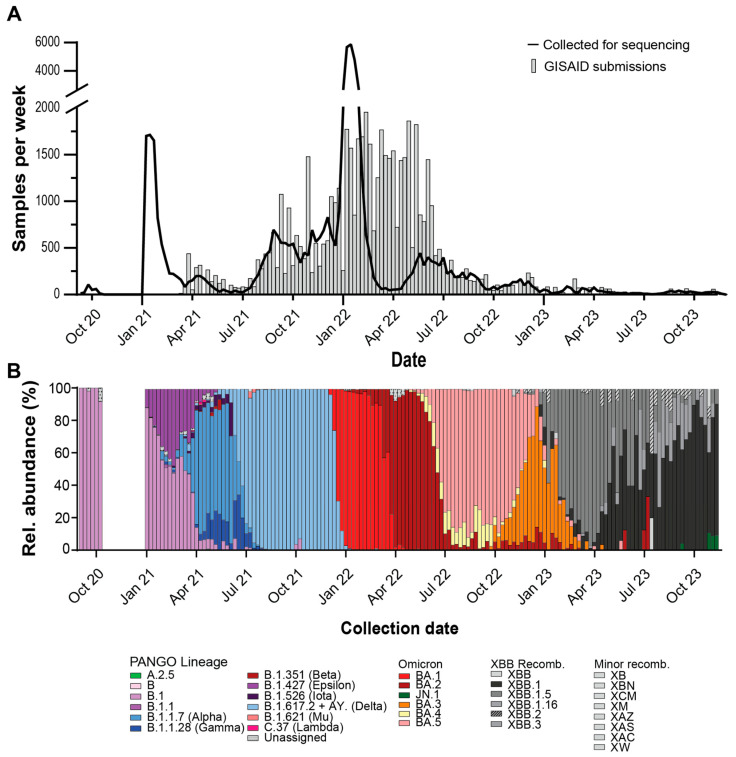
Whole viral genome sequencing of SARS-CoV-2 positive saliva samples. (**A**) Weekly RT-PCR positive samples (line) and weekly GISAID submissions (bars). (**B**) Relative weekly abundance of SARS-CoV-2 PANGO lineages. WHO VOC names in parentheses.

From September 2020 through February 2021, saliva and nucleic acid extracts from SARS-CoV-2 positive samples were stored at –80 °C until sequencing infrastructure was finalized. When NGS started in February 2021 through December 2021, weekly GISAID submissions generally matched RT-PCR positive sample collections (Figure 4A). In late December 2021 to February 2022, a surge of positive RT-PCR samples caused sample influx to exceed sequencing capacity. Over this period, samples collected on the most recent day were prioritized for WGS and others were banked for later sequencing. From February 2022 to May 2022, GISAID submissions greatly surpassed sample collections. During this period, banked samples were supplemented into NGS runs to meet sequencing capacity. After May 2022, GISAID submissions tracked sample collections and overall sample collections decreased compared to earlier dates.

Sample sequencing turnaround time, the time between participant sample registration and GISAID submission, is important to maintaining a ‘real-time’ analysis of circulating variants, therefore we analyzed the sample turnaround for our sequencing samples [33]. Turnaround time fluctuated over the surveillance period (Supplemental Figure S3). Samples collected and banked before sequencing infrastructure was established in February 2021 had the longest turnaround times. In May 2021 median turnaround times shortened to a desired 7–14 day turnaround time. Median turnaround times increased during December 2021 to February 2022, when sample collection exceeded sequencing capacity, but Q1 values remained within the 7–14 day window. When we compared our sequencing turnaround times to other sequencing laboratories in the USA, we observed that our median turnaround times were usually lower than weekly national medians. For our study and for other USA samples, 50% of weekly median turnaround times were below 12 (IQR = 9.0–19.75) and 23 (IQR = 19.0–30.0) days, respectively.

To observe trends in SARS-CoV-2 virus evolution, we looked at the relative abundances of each lineage using the GISAID lineage calls from our submitted samples over the surveillance period (Figure 4B). Major lineages reaching a majority relative abundance (>=50% of samples) in any week over the surveillance period were as follows: B.1, B.1.1.7 (Alpha), B.1.617.2 (Delta, with AY sublineages), BA.1 (Omicron), BA.2 (Omicron), BA.3 (Omicron), BA.5 (Omicron), XBB.1 (Omicron-recombinant), XBB.1.5 (Omicron-recombinant), XBB.1.16 (Omicron-recombinant). Notable lineages that were detected but did not meet majority abundance were A.2.5, B.1.1.28/P.1 (Gamma), B.1.351 (Beta), B.1.526 (Iota), B.1.621 (Mu), BA.4 (Omicron), C.37 (Lambda), and JN.1 (Omicron). The following non-XBB.1 recombinant lineages were detected during the surveillance period: XAC, XAS, XAZ, XB, XBB.2, XBB.3, XBN, XCM, XM, XW.

Finally, to assess the effect of viral load on sequencing performance, we compared TaqPath C_t_ values to consensus genome ambiguity (Supplemental Figure S4). We observed that fewer ambiguities were obtained at higher viral loads and ambiguity frequency rose as viral load decreased. Performing a LOWESS regression of all samples found that C_t_ values below 32.0 and 26.0 were associated with fewer than 30% and 5% ambiguities, respectively. We observed a low frequency (*n* = 998, 1.7%) of genomes with less than 30% ambiguity that failed pangolin lineage designation or GISAID acceptance. We also observed that a low proportion (*n* = 342, 0.6%) of consensus genomes with over 30% ambiguities were able to receive pangolin lineage designations and GISAID acceptance.

### 3.6. Diagnostic Test Failure Allowed Rapid Genotyping of Saliva Samples

**Figure 5:**
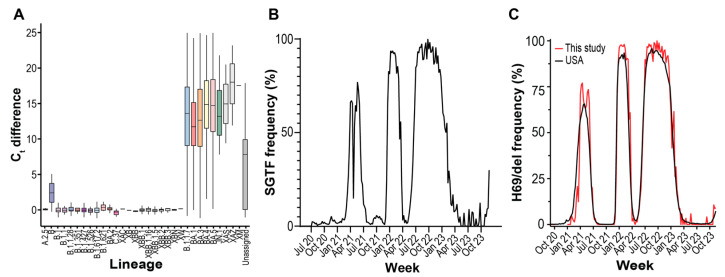
S gene target failure (SGTF) on TaqPath COVID-19 Combo Kit RT-PCR assays. (**A**) Difference between S gene and mean ORF1ab/N gene C_t_ values for PANGO variants displaying SGTF. The bottom, middle, and top of the boxes correspond to Q1, Q2, and Q3, respectively. Box whiskers correspond to the largest value no further than 1.5× IQR. (**B**) Weekly SGTF prevalence in local samples. SGTF is the S gene and mean ORF1ab/N gene C_t_ values differing by 3 or more. (**C**) Weekly abundance of samples in the GISAID database containing S:H69del mutation in USA and local samples.

The TaqPath COVID-19 Combo Kit detects the presence of SARS-CoV-2 genomes using primers and probes with homology to the ORF1ab, N, and S genes. When we compared the ORF1ab gene or N gene C_t_ values to S gene C_t_ values, we observed populations of genomes with discordant S gene C_t_ values (Figure 5A, Supplemental Figure S5). Genome sequencing revealed that this population was primarily composed of specific lineages. Of the major, notable, and recombinant lineages observed over the surveillance period (Figure 4B), we observed significantly large differences in median C_t_ values for B.1.1.7 (Alpha), BA.1 (Omicron), BA.3 (Omicron), BA.4 (Omicron), BA.5 (Omicron), JN.1 (Omicron), XAS, XAZ, and XM lineages compared to the B.1.1 lineage (Figure 5A; Tukey HSD, *p* < 0.005). Median ORF1ab C_t_ values differed between lineages but were not large enough to account for the observed differences (Supplemental Figure S6). The lineages displaying S gene target failure (SGTF) all contain the H69del/V70del mutations.

We detected three periods of high SGTF frequency over the surveillance period, as well as growing frequency at the end of the survey period (Figure 5B). We compared the frequency of our sequenced genomes containing H69/V70 deletion to the frequency of other USA genomes and observed that the mutation frequency patterns were identical to SGTF frequency (Figure 5C). Using WGS as reference, predicting H69del presence by SGTF using RT-PCR had a PPV = 0.98 (95% CI = 0.97–0.99) and NPV = 0.94 (95% CI = 0.94–0.96) (Supplementary Table S2). The depressed NPV is in part due to poor sequencing coverage of the H69 region due to the amplicon drop out caused by some Omicron lineages, and the subsequent inability to call the mutation during WGS [34]. Many of the false negatives received Omicron lineage designations by GISAID. In summary, multiple times during the surveillance period, RT-PCR diagnostic test failure enabled rapid H69/V70 deletion genotyping and facilitated a fast way to monitor changing lineage abundance.

## 4. Discussion

In this study we report on the multi-year study of monitoring the introduction and evolution of SARS-CoV-2 in Tempe, Arizona. Over 1.4 million RT-PCR assays were performed on saliva samples from over 360,000 participants. Over 94,000 samples tested positive for SARS-CoV-2 infection and over 69,000 samples were subjected to NGS to obtain whole SARS-CoV-2 consensus genomes. Over the survey period, we deposited over 54,000 SARS-CoV-2 consensus genomes into the GISAID database. Self-collected saliva samples were an adequate matrix for qualitative diagnosis of SARS-CoV-2 infection, and we obtained robust genome sequencing from most positive samples. Due to the robust results and the ease of sample collection, we believe self-administered saliva collection could provide an effective means for analyzing future respiratory disease outbreaks.

Our multi-year surveillance was primarily located in an urban metropolitan area of a major US city. Due to the high testing volume, high sequencing depth, and fast turnaround times, our surveillance program provided a robust representation of SARS-CoV-2 epidemiology [33]. Variant introduction and abundance patterns that we observed concur with patterns seen in other regional studies [35]. Upward and downward trends in testing frequency and positivity also appear consistent with regional (Figure 1C) and national [12] case counts and test positivity. However, in this study, we observe an overall increase in test positivity dynamics before and after infections peaked during the Omicron BA.1 variant introduction in January 2022 (Figure 2A). We also observed differences in test positivity between self-initiated testing and invited testing (Figure 3).

Changes in patterns of test positivity might be attributed to the motivations behind SARS-CoV-2 self-initiated testing. Testing motivations are multifaceted, involving personality, behavioral, clinical, and economic factors [36,37]. The availability of SARS-CoV-2 vaccines after December 2020 [38] may also be have contributed to altered testing motivations. Seroprevalence surveillance during this time period showed a large number of adults had antibodies to SARS-CoV-2 through vaccination, infection, or both [11]. Attitudes to testing may have been altered due to perceived protection status from vaccination or naturally acquired immunity (i.e., recent infection). Finally, the increased availability of commercial, at-home, SARS-CoV-2 tests may have altered the surveillance population and their motivations. Our study shows that while self-initiated and invited testing are generally in concordance, further understanding of testing motivations and how promotion of self-initiated testing may improve the representativeness of self-initiated testing.

Co-localizing RT-PCR and WGS operations resulted in valuable benefits. Close physical proximity reduced sample transit and storage time, minimized the number of freeze–thaw cycles a sample was exposed to, and facilitated communication between teams. Shipping time, storage time, and freeze–thaw cycles are all factors that negatively affect RNA stability [39]. Enhanced communication and coordination abilities allowed catching and correcting errors, as well as assisted in coordinating sequencing priority based on SGTF results.

In addition to internal communication, external guidance by state and federal agencies provided valuable information. The US Food & Drug Administration guidance documents assisted in the development of the Arizona Biodesign Clinical Testing Lab and establishing our surveillance program [40]. Federal agencies also organized regular status and discussion panels, such as the CDC’s Sequencing for Public Health Emergency Response, Epidemiology, and Surveillance (SPHERES) consortium [41]. These supplementary communication networks facilitated the prompt dispersal of information between testing sites, such as when novel variants displayed reduced sensitivity to diagnostic assays.

The genetic determinants of SGTF were known soon after the introduction of the Alpha variant [20]. The H69del/V70del mutation in the Spike protein provides no immune escape properties itself but rescues other Spike protein mutations with increased antibody evasion but impaired infectivity [42]. The increased prevalence of SGTF on SARS-CoV-2 specimens led to the design and adoption of TaqPath COVID-19 2.0 for primary diagnostic use. However, the continued use of the TaqPath COVID-19 Combo Kit Assay continued to provide important public health data. Broadly, it allowed facilities without sequencing capabilities to monitor variant introduction [43]. SGTF provided fast variant typing during the Alpha-Delta, Delta-Omicron BA.1, Omicron BA.1-Omicron BA.2, Omicron BA.2-Omicron BA.3/4/5, and Omicron BA.3/4/5-XBB transitions (Figure 4B and Figure 5A). For sequencing laboratories, SGTF was useful when sample positivity exceeded sequencing capacity, as it allowed researchers to direct sequencing efforts towards samples suspected of being newly introduced variants. Subsequent WGS allowed the confirmation of those variants and allowed surveillance for novel mutations within introduced lineages.

Although most samples with low genome ambiguity were accepted for database submission, we observed a small number (*n* = 979, 1.4%) of samples that had high genome completeness (<30% ambiguities) but failed lineage designation or GISAID submission (Supplemental Figure S4). Samples with contamination or true co-infection may have received mixed-lineage designations and been excluded or denied database submission. There may also be some cases where a sample contained ambiguities at nucleotides critical in lineage designation, or samples belonged to undesignated lineages at the time of analysis, such as recombinant lineages. Amplicon-based sequencing was the primary sequencing method, therefore mutations in viral genomes located within primer binding regions would cause reduced sequencing coverage of that amplicon. This phenomenon, termed amplicon drop-out, has occurred multiple times over the course of SARS-CoV-2 evolution [44,45]. Samples with ambiguities in positions critical to lineage-defining specificity may have been incorrectly assigned a lineage or left unassigned, causing them to fail submission.

Our results align with a previously published epidemiological study using self-collected saliva samples which showed saliva had low rates of invalid tests, good genome coverage recovery, and good variant typing rates [46]. Highlighting the world-wide scale of the COVID-19 pandemic, lineage abundance dynamics in Arizona, the USA, and throughout the world are commonly characterized by wave-like periods dominated by a single or few lineages [7,47]. However, it is difficult to directly compare surveillance programs, as even within saliva-based testing, surveillance programs can differ in their initial detection assays (RT-PCR, RT-LAMP), sample processing (with/without RNA extraction), sequencing methodology (amplicon, hybrid enrichment), and sequencing platform (Illumina, Oxford Nanopore), all of which influence surveillance sensitivity and cost [14,34,48,49]. Like other studies [17], we did observe a higher limit of detection of SARS-CoV-2 in saliva than in NPS samples, so we recommend that NPS remain the gold-standard for diagnostic, point-of-care testing and critical applications.

Since most of our surveillance derived from samples self-collected from participants who self-initiated testing, our program and its results come with limitations and caveats. Our cohort skews toward college-aged participants (21%, 18–21 years). While our study shows good concordance with state case rates (Figure 2C), college students may have attitudes, behaviors, and schedules that contribute to differences in disease spread compared to other groups [50]. Our cohort also contained pediatric and youth participants (9.1%, 0–17 years). Instructions for sample collection did not include special instructions for these participants. Future studies and disease surveillance programs could include additional instructions or collection methods for pediatric participants, although further research in this area is still needed (reviewed in [51]). The single collection methodology used in our study may also restrict our surveillance of people with physical or developmental disabilities that complicate sample collection.

Ultimately, in this study we determined that self-collected saliva-based surveillance assays can provide important epidemiological information during a public health crisis. We observed that our surveillance system (i) provided an effective means to broadly assess community health and epidemic status, (ii) provided a means to effectively detect outbreaks within the population to target remediation strategies, and (iii) quickly provided high-quality material to enable the monitoring of pathogen evolution by WGS during an epidemic crisis. Due to its simple collection and low invasiveness, continued examination of saliva as a testing matrix for routine and pandemic pathogen surveillance is important.

## Figures and Tables

**Table 1:** Limit of detection for SARS-CoV-2 virus particles in a saliva matrix.

Assay	Virus Particles/mL Saliva	Positive/ Total	Average C_t_ Value (St. dev.)			
			RNAse P	ORF1ab	N Gene	S Gene
Limit of detection (LOD)	100,000,000	3/3	21.81 (0.1)	18.31 (0.16)	18.84 (0.17)	18.36 (0.12)
	50,000,000	3/3	21.76 (0.1)	19.46 (0.17)	19.88 (0.06)	19.44 (0.12)
	25,000,000	3/3	21.7 (0.22)	20.45 (0.22)	20.75 (0.06)	20.34 (0.13)
	12,500,000	3/3	21.71 (0.19)	21.4 (0.2)	21.67 (0.18)	21.29 (0.09)
	6,250,000	3/3	21.72 (0.19)	22.14 (0.35)	22.55 (0.12)	22.01 (0.35)
	3,125,000	3/3	21.61 (0.13)	23.11 (0.16)	23.56 (0.19)	23.13 (0.21)
	1,562,500	3/3	21.65 (0.04)	24.16 (0.2)	24.46 (0.08)	24.15 (0.18)
	781,250	3/3	21.63 (0.2)	25.28 (0.36)	25.42 (0.19)	25.3 (0.27)
	390,625	3/3	21.68 (0.12)	25.83 (0.2)	26.39 (0.1)	25.89 (0.18)
	195,313	3/3	21.67 (0.11)	27.04 (0.11)	27.48 (0.1)	27.25 (0.07)
	97,656	3/3	21.53 (0.19)	27.59 (0.27)	28.38 (0.16)	27.84 (0.48)
	48,828	3/3	21.53 (0.1)	28.35 (0.57)	29.93 (0.81)	29.76 (0.92)
	24,414	2/3	21.49 (0.25)	30.9 (2.39)	31.99 (0.82)	31.3 (0.77)
	12,207	3/3	21.5 (0.39)	31.68 (0.48)	37.3 (0.82)	35.49 (2.09)
	6103	1/3	21.62 (0.04)	35.15	-	36.65
	3052	0/3	21.78 (0.21)	-	-	-
Confirmation	12,500 (1× LOD)	24/24	22.89 (0.22)	30.36 (0.61)	30.78 (0.49)	30.54 (0.75)
	25,000 (2× LOD)	24/24	22.71 (0.26)	29.35 (0.54)	29.83 (0.55)	29.38 (0.73)
	50,000 (4× LOD)	24/24	22.68 (0.36)	28.03 (1.3)	28.5 (1.36)	28.08 (1.43)
	100,000 (8× LOD)	24/24	22.48 (0.7)	26.91 (1.49)	27.65 (0.71)	26.8 (2.17)
	200,000 (16× LOD)	24/24	22.85 (0.19)	26.25 (0.51)	26.89 (0.24)	26.08 (0.54)
	400,000 (32× LOD)	24/24	22.78 (0.29)	25.14 (0.92)	26.01 (0.38)	25.03 (0.88)

**Table 2:** Comparison of nasopharyngeal swab (NPS) and saliva sampling method for detection of SARS-CoV-2 infection using the TaqPath COVID-19 Combo Kit qPCR assay.

		NPS	
		Positive	Negative
Saliva	Positive	19	1
	Negative	1	127
	Total	20	128

**Table 3:** SARS-CoV-2 RT-PCR and NGS assays performed from April 2020 to December 2023.

	No.	%
Total tests	1,434,873	
Negative tests	1,329,621	92.7%
Positive tests	94,330	6.6%
NGS sequenced	69,595	73.8% ^1^
Deposited into GISAID	54,040	77.6% ^2^
Invalid tests	10,922	0.8%

^1^ Percentage of RT-PCR positive samples. ^2^ Percentage of NGS sequenced samples.

**Table 4:** Demographics of surveillance participants from April 2020 to December 2023.

Characteristics	No.	(%)
Total unique participants	366,681	
Sex		
Male	186,090	(50.7)
Female	176,649	(48.2)
Unknown/Invalid or no answer	3942	(1.1)
Race		
White	202,398	(55.2)
Black or African American	20,742	(5.7)
Asian	16,355	(4.5)
American Indian or Alaska Native	13,428	(3.7)
Native Hawaiian or other Pacific Islander	1435	(0.4)
Other	34,149	(9.3)
Unknown/Declined/Invalid or no answer	77,957	(21.3)
Ethnicity		
Not Hispanic or Latino	225,704	(61.6)
Hispanic or Latino	80,244	(21.9)
Unknown/Declined/Invalid or no answer	60,733	(16.6)
Age at first test		
0–17	33,346	(9.1)
18–21	77,053	(21)
22–29	74,638	(20.4)
30–39	64,335	(17.5)
40–49	45,621	(12.4)
50–59	36,209	(9.9)
60+	34,486	(9.4)
Invalid or no answer	993	(0.3)

## Data Availability

Viral genome sequences generated in this surveillance program were submitted to the GISAID nCov database and can be obtained using the EPI_SET ID: EPI_SET_250224ka. RT-PCR results, sequencing results, and patient metadata is available at Data Dryad: https://doi.org/10.5061/dryad.z08kprrsh.

## Supplementary Materials

The following supporting information can be downloaded at https://www.mdpi.com/article/10.3390/diagnostics15202663/s1. Figure S1: Cross- and autocorrelation analyses of surveillance RT-PCR test positives and Arizona COVID-19 positive cases. Figure S2: Participant counts of self-initiated and invited testing programs. Figure S3: GISAID collection to submission turnaround time for surveillance program and other USA samples. Figure S4: TaqPath C_t_ values and consensus genome ambiguity of saliva samples. Figure S5: S gene target failure on TaqPath COVID-19 Combo Kit RT-PCR assays. Figure S6: TaqPath COVID-19 Combo Kit ORF1ab C_t_ values of saliva samples by SARS-CoV-2 lineages; Table S1: Limit of detection analysis of SARS-CoV-2 detection in nasopharyngeal swabs using the TaqPath COVID-19 Combo Kit qPCR assay. Table S2: Predictive power of RTPCR S gene target failure to predict presence of H69del in saliva samples.

## Abbreviations

The following abbreviations are used in this manuscript:

(S)GTF	(S) Gene Target Failure
CDC	Centers for Disease Control and Prevention
COVID-19	Coronavirus disease 2019
EUA	Emergency Use Authorization
GISAID	Global Initiative on Sharing All Influenza Data
HIPAA	Health Insurance Portability and Accountability Act
LOWESS/LOESS	Locally Weighted/Estimated Scatterplot Smoothing
NGS	Next-generation sequencing
NPA	nasopharyngeal aspirate
NPS	nasopharyngeal swab
PANGO(LIN)	Phylogenetic Assignment of Named Global Outbreak (Lineages)
RT-PCR	reverse transcriptase polymerase chain reaction
SARS-CoV-2	Severe acute respiratory syndrome coronavirus 2
VOC	Variant of Concern
VOI	Variant of Interest
VUM	Variant Under Monitoring
WGS	Whole Genomes Sequencing
WHO	World Health Organization
