# Supplementary material for: Large-Scale Saliva-Based Clinical Surveillance Enables Real Time SARS-CoV-2 Outbreak Detection and Genomic Tracking (Arizona, 2020–2023)
Source: Diagnostics (Basel). 2025 Oct 21;15(20):2663. doi: 10.3390/diagnostics15202663 (PMC12564657; doi:10.3390/diagnostics15202663)
Supplement: Supplementary file 1 [file diagnostics-15-02663-s001.zip › diagnostics-3861618-supplementary.pdf]

Supplementary Materials:

Large-Scale Saliva-Based Clinical Surveillance Enables Real Time SARS-CoV-2 Outbreak Detection and Genomic Tracking (Arizona, 2020–2023)

Steven C. Holland [1], ABCTL Diagnostic Testing and Sequencing Teams [1,2,†], Ian Shoemaker [1], Theresa Rosov [1], Carolyn Compton [1], Joshua LaBaer [1], Efrem S. Lim [3] and Vel Murugan [1,4,5,*]

1 Virginia G. Piper Center for Personalized Diagnostics, Biodesign Institute, Arizona State University,
Tempe, AZ 85281, USA; schollan@asu.edu (S.C.H.); shoemaker.ian@gmail.com (I.S.); trosov@cndlifesciences.com (T.R.); ccompto3@asu.edu (C.C.); jlabaer@asu.edu (J.L.)

2 ASU Biodesign Clinical Testing Laboratory, Biodesign Institute, Arizona State University, Tempe, AZ 85281, USA

3 Communicable Diseases Agency, Singapore 307684, Singapore; efrem_lim@cda.gov.sg

4 College of Health Solutions, Arizona State University, Phoenix, AZ 85004, USA

5 Health Observatory, Arizona State University, Phoenix, AZ 85004, USA

* Correspondence: vel.murugan@asu.edu; Tel.: +1-480-727-0402

† ABCTL Diagnostic Testing and Sequencing Teams is provided in the Acknowledgments.

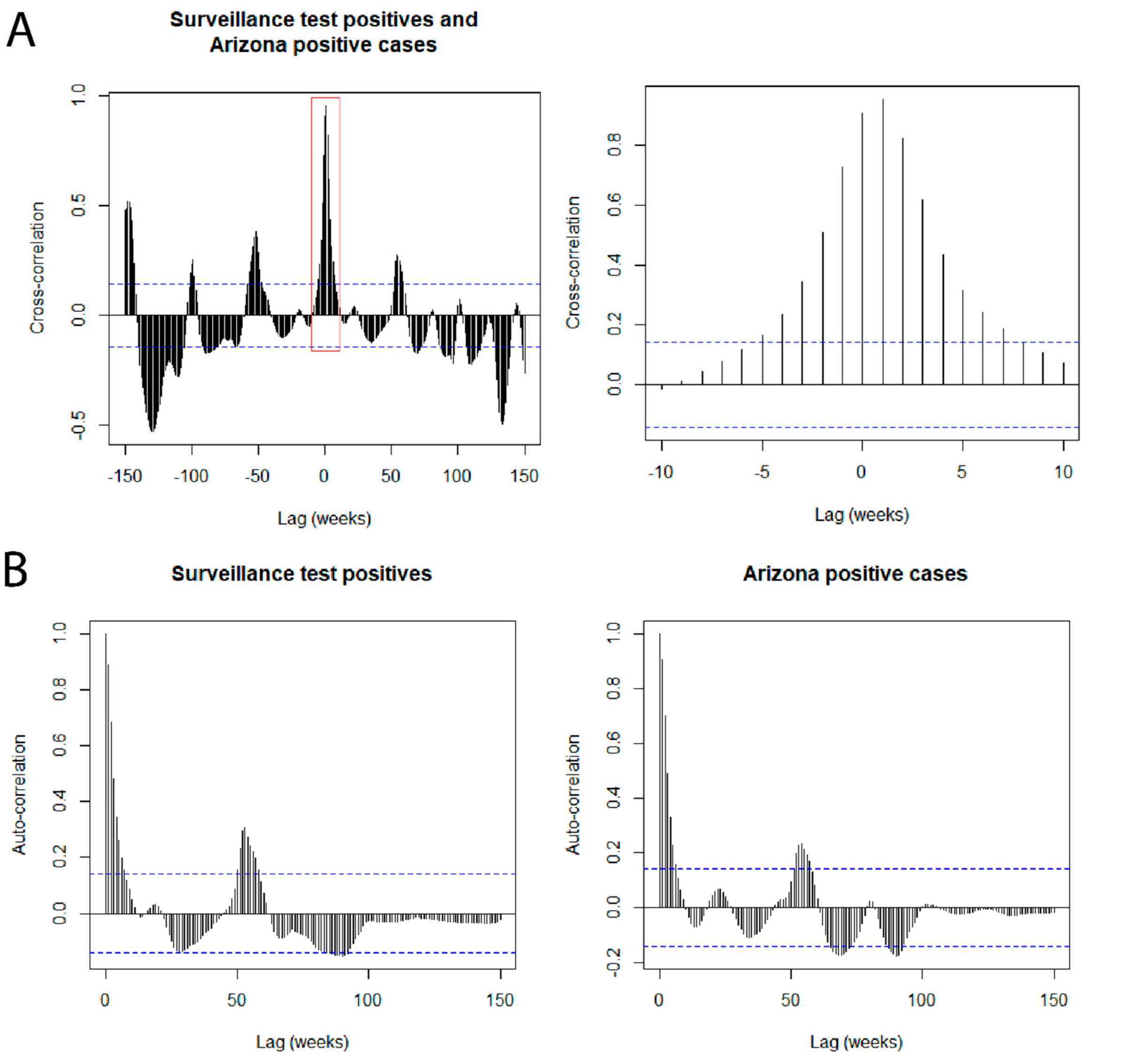

Supplementary Figure S1: Cross- and autocorrelation analysis of surveillance RTPCR test positives and Arizona COVID-19 positive cases. Blue dashed lines indicate 95% confidence intervals. (a) Left panel: Cross-correlation analysis of surveillance test positives and Arizona positive cases. Right panel: Expanded view of red region in left panel. (b) Left panel: Autocorrelation analysis of surveillance test RTPCR test positives. Right panels: Autocorrelation analysis of Arizona positive cases.

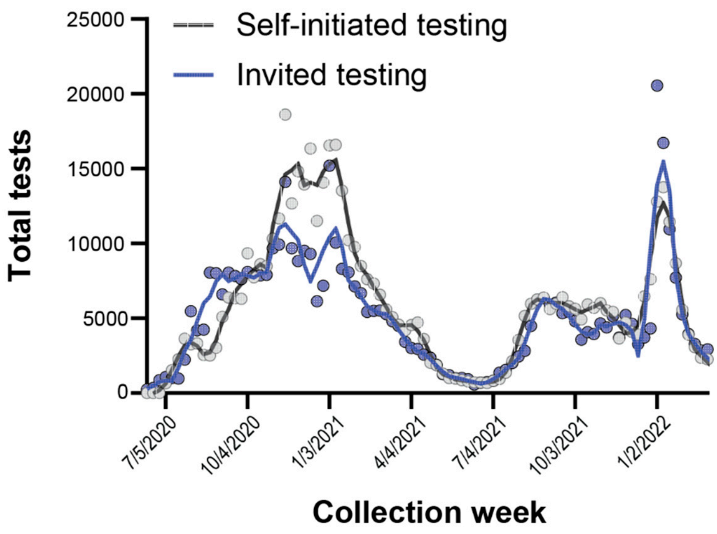

Supplementary Figure S2: Participant counts of self-initiated and invited testing programs.

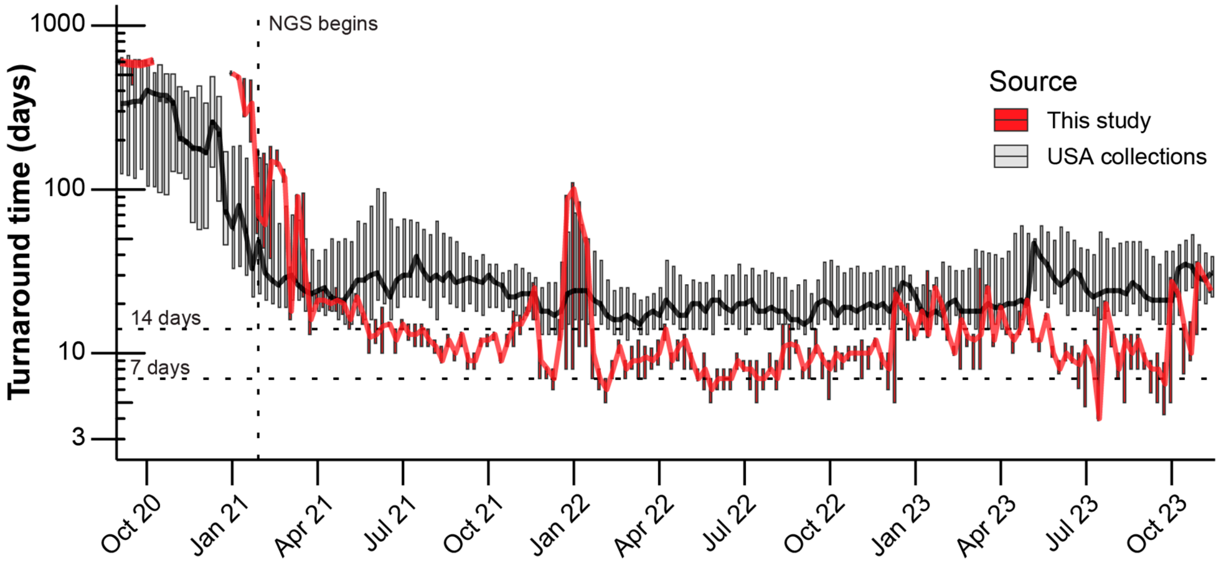

Supplementary Figure S3: GISAID collection to submission turnaround time for surveillance program and other USA samples. Solid line corresponds to median turnaround time. Bottom and top of boxes correspond to Q1 and Q3 values, respectively.

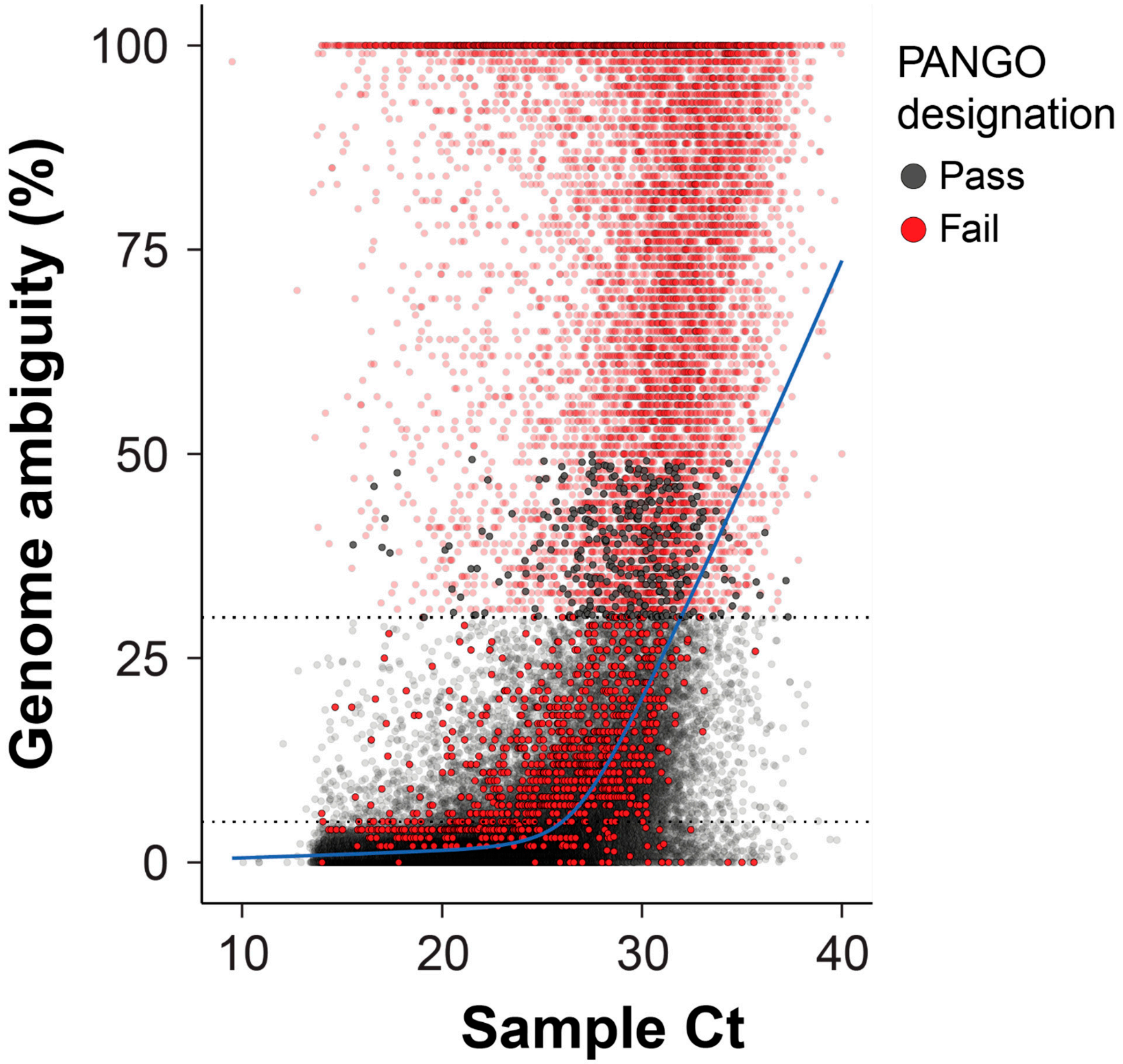

Supplementary Figure S4: TaqPath C_t values and consensus genome ambiguity (percent of non-A,T,C,G nucleotides in consensus genome) of saliva samples. Blue line shows LOWESS smoothing of all samples.

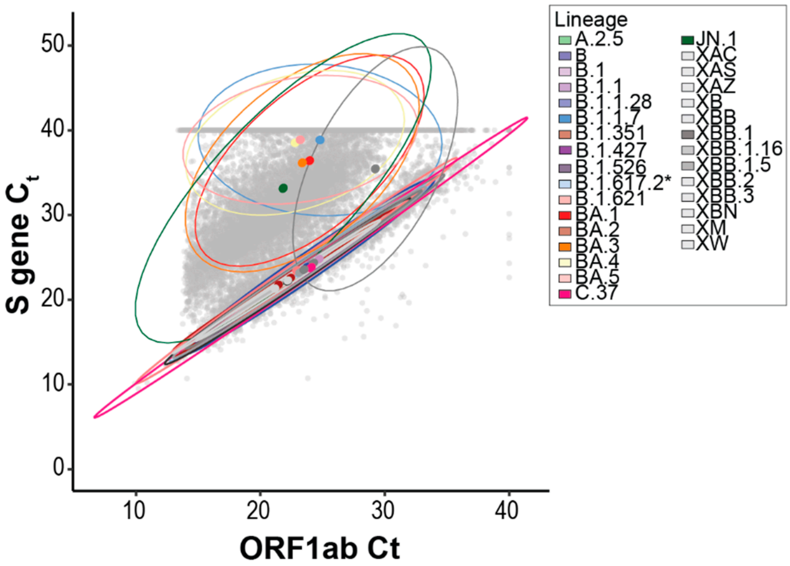

Supplementary Figure S5: S gene target failure (SGTF) on TaqPath COVID-19 Combo Kit RT-PCR assays. Mean ORF1ab/N gene C_t values and S gene C_t values of positive saliva samples. Ellipses capture 95% of samples belonging to each PANGO variant. Colored points indicate ellipse centroid.

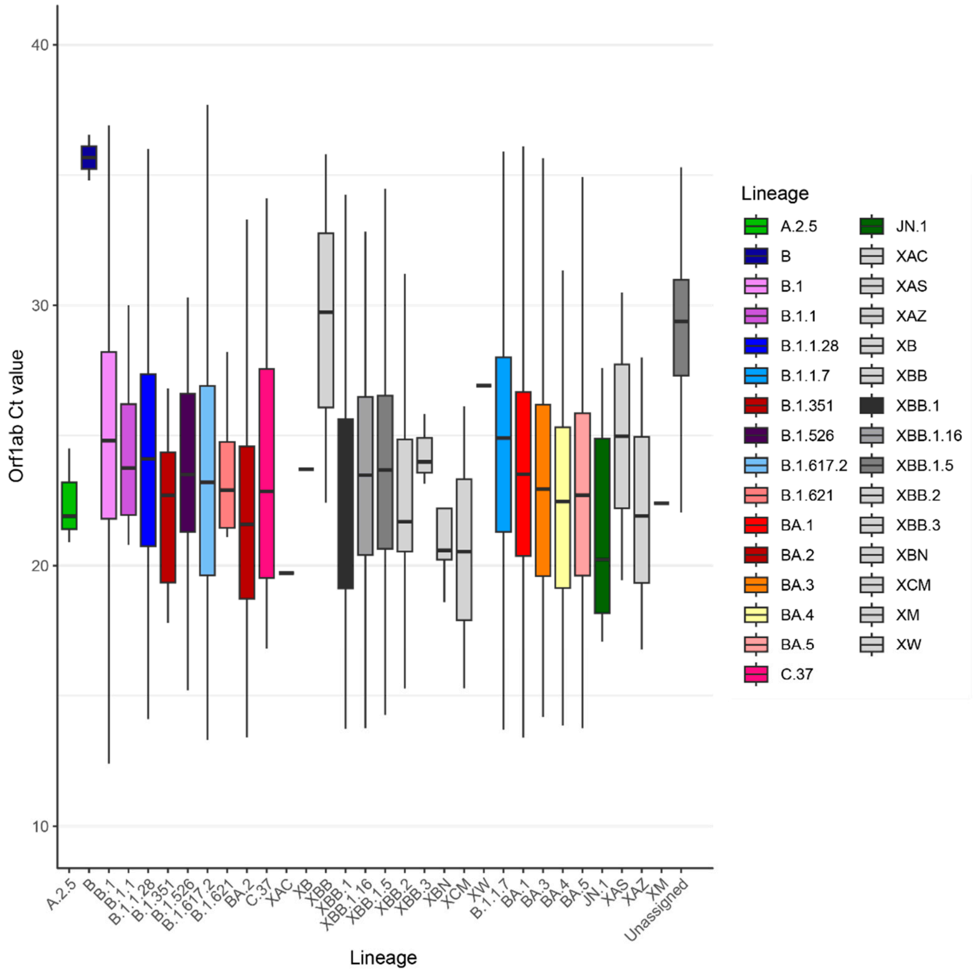

Supplementary Figure S6: TaqPath COVID-19 Combo Kit Orf1ab Ct values of saliva samples by SARS-CoV-2 lineages.

Table S1: Limit of detection analysis of SARS-CoV-2 detection in nasopharyngeal swabs using the TaqPath COVID-19 Combo Kit qPCR assay.

Assay	Virus particles/mL VTM	Positive /Total	Average C_t value (St. dev.)			
			RNAse P	ORF1ab	N gene	S gene
Limit of detection (LOD)	500,000	3/3	28.00 (0.22)	21.73 (0.3)	22.69 (0.28)	19.34 (0.77)
	50,000	3/3	27.73 (0.57)	25.55 (0.25)	26.26 (0.19)	22.77 (0.81)
	5,000	3/3	27.8 (0.07)	26.85 (2.23)	28.66 (0.88)	25.27 (2.02)
	1,000	3/3	27.53 (0.24)	33.16 (1.46)	32.24 (0.7)	32.87 (1.52)
	500	1/3	27.47 (0.03)	33.26	34.12	33.72
	250	0/3	26.87 (0.73)	-	-	-
Confirmation	1,000 (1x LOD)	24/24	24.51 (0.97)	31.25 (3.31)	31.37 (6.15)	29.6 (5.04)
	2,000 (2x LOD)	24/24	24.63 (0.15)	31.22 (2.04)	32.68 (1.08)	28.74 (3.16)
	3,000 (3x LOD)	24/24	24.13 (0.86)	25.63 (2.89)	29.49 (2.98)	24.74 (3.65)
	5,000 (5x LOD)	24/24	24.93 (0.14)	29.11 (0.81)	30.98 (0.62)	26.13 (2.8)

Table S2: Predictive power of RTPCR S gene target failure to predict presence of H69del in saliva samples.

		RT-PCR SGTF	
		Positive (SGTF)	Negative (no SGTF)
WGS	Positive (H69del)	26,737	1,138
	Negative (H69)	520	21,437
	Total	27,257	22,575

Positive predictive value = 0.98 (95% CI = 0.97-0.99)

Negative predictive value = 0.94 (95% CI = 0.94-0.96)
